# Mechanisms of cardiomyocyte dysfunction induced by extracellular histones

**DOI:** 10.1038/s41420-026-03274-w

**Published:** 2026-07-27

**Authors:** Moza M. Al-Owais, Zhaokang Yang, Derek S. Steele, Arun V. Holden

**Affiliations:** School of Biomedical Sciences, Faculty of Biological Sciences, University of Leeds, Leeds, UK

**Keywords:** Physiology, Cardiovascular biology

## Abstract

Circulating histones released from damaged cells cause secondary impairment to organs, including the heart. Here, we investigate their effects and the underlying pathological mechanisms on isolated ventricular cardiomyocytes. Exogenous H3 or H4 (0.01–1 µg ml^−1^) induced progressive membrane depolarization, action potential prolongation, membrane potential oscillations resembling early afterdepolarizations, and Ca^2+^ dysregulation. These changes resulted from a histone-induced nonspecific depolarizing current with a linear current voltage relationship, consistent with the formation of transient pores in the sarcolemma. These effects were replicated by the addition of endogenous H3 isolated from media surrounding myocytes where necrosis has been induced. Acetylation by histone deacetylase inhibitors improved cell viability, reduced and delayed the electrophysiological changes observed with nonacetylated H3. Recombinant pan-acetyl H3 eliminated lethal depolarization. Complexes of histone and histone antibodies produced smaller depolarizations after longer exposures and delayed electrophysiological response. This is consistent with neutralization of the positive charges associated with the histone and suggest a novel therapeutic strategy to limit pathology resulting from histone toxicity.

## Introduction

Histones, the building block of chromatin, consist of four core histones—H2A, H2B, H3 and H4—and package DNA into a string of nucleosomes that compacts chromosomes, and regulate their stability, DNA repair and gene transcription and expression [1,2]. Histones have activities outside the nucleus, regulating cellular metabolism, and extracellular histones are antimicrobial, can modulate and enhance inflammatory responses, and can be cytotoxic [3]. Chromatin structure can be altered by oxidative stress, thus damaging histones directly, histones can leak from damaged cells [4,5] or be released from neutrophils as part of the response to inflammation [6]. Clinical studies show that histones are released into the circulation following events produced by tissue damage [7] at levels between 10 and 230 µg ml^−1^, and circulating plasma histone levels correlate with outcomes [8–10].

Circulating histones have also been linked to diastolic dysfunction [11,12], potentially worsening septic myocardial injury, and may trigger myocyte apoptosis [13]. An increase in circulating histones correlated with increased levels of the cardiac troponin-T biomarker of cardiomyocyte death, and the Sequential Organ Failure Assessment scores and mortality in sepsis patients [14,15]. In a subset of patients, without prior cardiac disease, increased histone levels correlated with left ventricular dysfunction and arrhythmias [14].

In vivo studies using a mouse model showed that injection of histones 25–75 µg g^−1^ produced multiple organ failure, with different organs failing at different times, with 75 µg g^−1^ resulting in 25% mortality within 15 min [15]. These effects have been reproduced in mouse models of sepsis [16–18]. Histones accumulate more rapidly in the heart than in other organs. Moreover, fluorescein-labelled histones have been shown to bind to the myocardial cell membranes before becoming internalized [14,16]. Following a sepsis-induced increase in circulating histones, elevated cardiac troponin-T, left ventricular contractile dysfunction and ventricular arrhythmias were developed [12,19].

In vitro cellular studies demonstrate extra-nuclear, intracellular actions of histones-DNA damage is signalled by the linker histone H1.2 and triggers apoptosis *via* mitochondria [20], and H3 and H4 binding to endosomal Toll-Like Receptors (TLRs) produces pro-inflammatory cytokines [21]. Actions of extracellular histones can be mediated by entry by endocytosis, binding to surface membrane receptors TLR2 and TLR4, and enhanced by oxidative stress [22]. The high positive charge of the histone molecules allows the tight packing of DNA in the nucleosome, and its modification by acetylation and methylation allow modulation of expression. The cascade of cellular and tissue inflammatory responses to extracellular histones can be reduced by histone acetylation and methylation, and by negatively charged molecules [23–25].

In fatal cases of trauma, tissue damage and sepsis, ventricular arrhythmia provides the final common path to death of the individual. Although cardiac arrhythmia is irregular spatio-temporal propagating activity at a ~10 cm, > 5 min, tissue and organ level, their triggers can be quantitatively understood from the electrophysiology of isolated cardiomyocytes.

Here, we examined the effects of a range of histone (H3 and H4) concentrations (0.001–1 µg ml^−1^) on the electrophysiology (action potentials and intracellular calcium activities) of isolated cardiomyocytes, to quantitatively investigate histone-induced cell damage, and how they may be ameliorated, to lead towards a possible therapeutic strategy.

## Results

### Histones caused rapid membrane disruption in adult rat ventricular cardiomyocytes in a concentration and time dependent manner

**Fig. 1:**
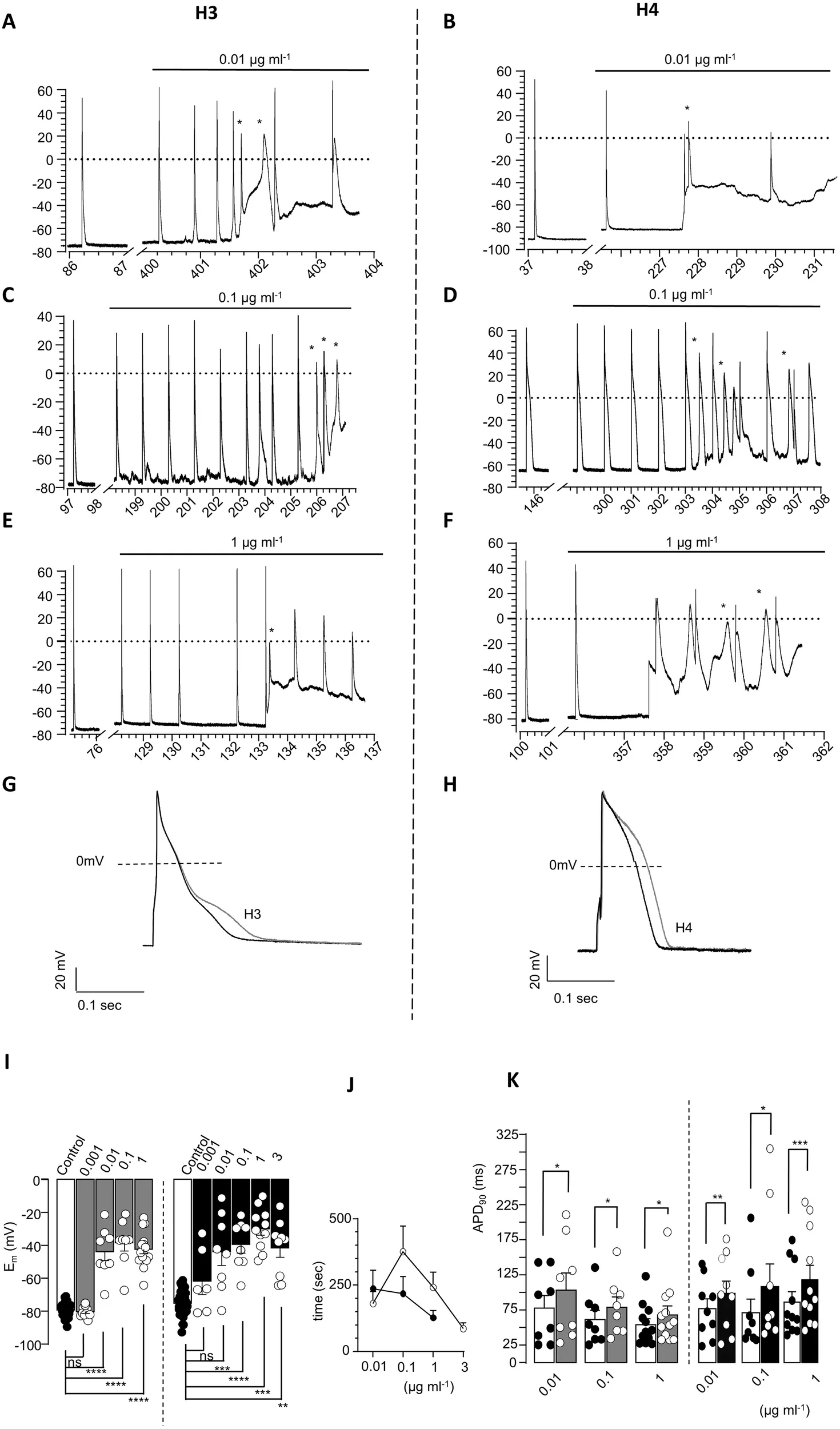
Histones destabilise and disrupt rat ventricular myocytes membrane potential in concentration and time dependent manner. Representative recordings of triggered APs before and during (horizontal bar) superfusion of histone proteins and the emergence of histone evoked depolarisations, spontaneous APs, and EADs with commercially obtained H3 (**A**, **C**, **E**) or H4 (**B**, **D**, **F**) at different concentration. Spontaneous APs marked with Asterisk symbol. **G**, **H** Superimposed illustrative triggered APs showing prolongation of APD; controls (black) and H3 or H4 at concentration of 0.01 µg ml^−1^ (grey). **I** summary data of measured *E*_m_ before (control; white) and during superfusion of different concentration of H3 (grey), or H4 (black; *P* values obtained from one way ANOVA with Bonferroni’s post hoc test), in rat ventricular myocytes. **J** Cumulative time for loss of *E*_m_ following superfusion of histone proteins (H3; filled symbol, H4, open symbol) (**K**) Cumulative APD_0.9_ summary in control (white) and during superfusion of histone proteins (H4; black, H3; grey; *P* values obtained from a two-tailed paired Student’s *t*-test). Data shown as Mean ± SEM, each circular symbol represents individual data point, *P < 0.05, **P < 0.01, ***P < 0.001, ****P < 0.0001.

To provide a quantitative approach to study histones effects on cardiomyocyte membrane behaviour, commercially sourced H3 or H4 histones were applied *via* bathing solution (0.001–1 µg ml^−1^) and consequent effects on action potentials (AP) recorded using whole cell current clamp. Figure 1 shows examples of membrane potential (*E*_m_) recording obtained from 6 myocytes, where APs were periodically evoked every 1 s, before and during superfusion of either H3 (Fig. 1A, C, E) or H4 (Fig. 1B, D, F), at concentrations from 0.01 to 1 µg ml^-1^. The mean ± SEM diastolic E_m_ in control conditions was −77.1 ± 0.67 (*n* = 83 cells) taken from 29 separate isolations. Following H3 or H4 application (0.01–1 µg ml^-1^), there was a slow gradual depolarisation of the E_m_ leading to a burst of spontaneous APs with reduced maximum rate of depolarisation, prolonged action potential durations (APD; Fig. 1G, H), and early after depolarization-like (EADs) oscillations as shown in the time series representative trace examples (Fig. 1A–H). The EADs-like oscillations effects were seen in 77% of cells examined during the application of H3 and in 40% of the cells examined during the application of H4. The mean ± SEM of histones concentration dependence on *E*_m_ depolarisation is shown in Fig. 1I; note that a concentration of 0.001 µg ml^−1^ did not differ significantly from control (Supplementary Fig. 1). Fig. 1J shows exposure time for the depolarizing shift to occur at different concentration, and Fig. 1K shows summary mean ( ± SEM) APD measured at 90% (APD_90_) of repolarization in control and during superfusion of H3 or H4.

### Histones induced Ca^2+^ dysregulation in cardiomyocytes

**Fig. 2:**
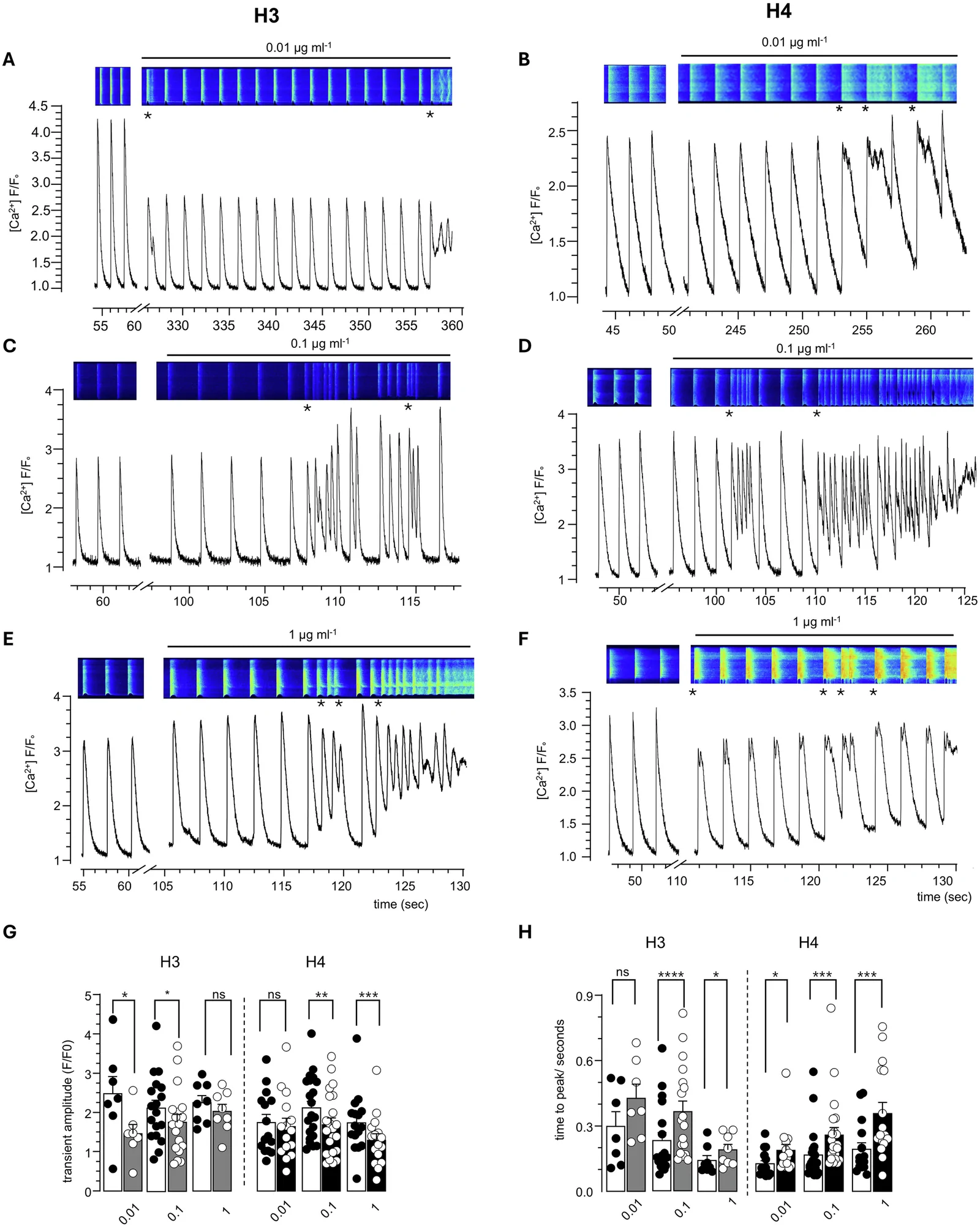
Histones proteins disrupt intracellular Ca^2+^ handling in rat ventricular myocytes. **A**–**F** selected examples of linescan images of steady-state Ca^2+^ transients train elicited by 0.5/s field stimulation (upper panels), and the Ca^2+^ profile signals associated (lower panels) shows dynamic change of Ca^2+^ with time both in control conditions and during (horizontal bar) the application of either H3 (**A**, **C**, **E**) or H4 (**B**, **D**, **F**) at concentrations indicated. **G**, **H** Ca^2+^ transients cumulative data in control (white) and during superfusion of H3 (grey), or H4 (black; paired *t*-test). Each circular symbol represents individual data point; *P* values obtained from a two-tailed paired Student’s *t*-test, *P < 0.05, **P < 0.01, ***P < 0.001, ****P < 0.0001.

To investigate whether the electrophysiological effects shown in Fig. 1 were associated with changes in intracellular Ca^2+^ regulation [Ca^2+^]_i_, cells were loaded with fluo-4 AM and field stimulated at 0.5/s during line-scan confocal Ca^2+^ imaging, before and during exposure to H3 or H4. Figure 2 shows representative line-scan images from 6 cardiomyocytes, and below each, the corresponding line profile showing [Ca^2+^]_i_ transients before and during superfusion of H3 or H4. Under control conditions, the [Ca^2+^]_i_ transients appear uniform in amplitude and time course; the % coefficient of variation of half-width of these events were 3.2% (*N* = 30), 5.9% (*N* = 25), 6.7% (*N* = 41), 7.1% (*N* = 29), 2.5% (*N* = 28), and 3.8% (*N* = 27) respectively, for the examples shown in Fig. 2A–F. Following H3 (Fig. 2A, C, E) or H4 (Fig. 2B, D F) exposure changes in [Ca^2+^]_i_ transients occurred including the emergence of spontaneous [Ca^2+^]_i_ transients, increased variability in transient amplitude, prolongation of triggered [Ca^2+^]_i_ transients, increase in the resting [Ca^2+^]_i_, and in some cases, oscillations resembling EADs before the loss of responses to field stimulation.

Figure 2G shows the mean ± SEM of H3 and H4 effects on the amplitude of triggered Ca^2+^ transients. The effect of H3 and H4 on the time to peak of the transients is shown in Fig. 2H.

The characteristic sequence of effects of H3 or H4 was seen at concentrations from 0.01 to 1 µg ml^−1^. Two-thirds of cells examined during the application of either H3 (*n* = 39) or H4 (*n* = 59) exhibited irregular triggered intracellular Ca^2+^ transients and spontaneous Ca^2+^ oscillations.

The line-scan fluorescence images show that almost all the spontaneous transients were associated with a rapid and spatially uniform initial activation phase, like that induced by field stimulation. This suggests that the events reflect spontaneous APs, rather than intracellular Ca^2+^ waves secondary by SR Ca^2+^ overload.

### Histones induce a non-selective sarcolemmal leak and increased membrane voltage noise in cardiomyocytes

The results of Fig. 1 directly reflect histone induced changes in ionic currents involved in the generation of the AP, while Fig. 2 indicates the involvement of Ca^2+^ ions, perhaps *via* membrane currents; the voltage dependent Ca^2+^ channels, or via permeation of *I*_Na+_ channels. Selective blockers of these channels were used to reduce their influence on [Ca^2+^]_i_. Current voltage relations were generated rapidly *via* a ramp clamp protocol designed to elicit both *I*_Na+_ and *I*_Ca2+_ currents in cardiomyocytes, then blockers were added; A803467 has been shown to inhibit cardiac *I*_Na+_ 1.5 channels [26], thus preventing Ca^2+^ permeation [27], and nifedipine to inhibit cardiac L-type *I*_Ca2+_ channels, thus blocking Ca^2+^ influx and subsequently reducing cytosolic Ca^2+^ concentrations [28].

**Fig. 3:**
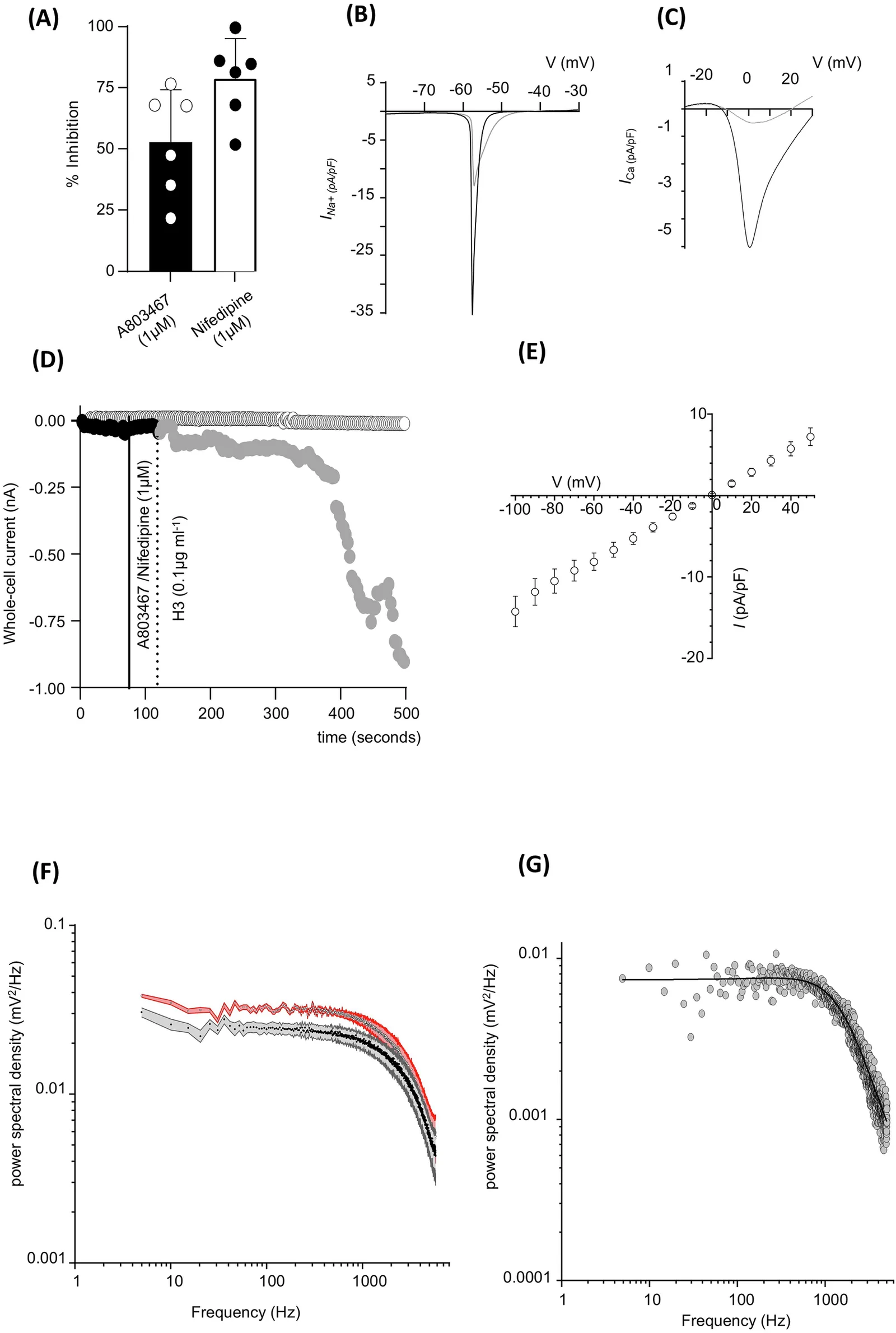
H3 induction of non-specific leak and membrane damage. **A** Mean ± SEM data showing the % of inhibition of Native *I*_Nav_ and *I*_CaL_ recorded in rat ventricular cardiomyocytes using A803467 and Nifedipine obtained via ramp voltage increasing from −100 to +60 mV over 500 ms. Representative scaled ramp-generated Native *I*_Nav_ and *I*_CaL_ currents traces shown in (**B**, **C**) both in control (black) and during superfusion (grey) with these inhibitors. **D** Whole-cell current time series recordings obtained from two myocytes. Both cells are superfused with *I*_Na_ 1.5 and *I*_Ca_ L-type channels blockers added at the time indicated by the solid vertical line. Upper (open circles); a representative time series obtained from control experiment without H3 superfusion, illustrating the recording stability and no development of leak current following application of channel blockers. Lower (black and grey symbols); a representative time series example, where H3 (0.1 µg ml^−1^) was added at time indicated by arrowhead and dashed vertical line, the leak develops slowly for 300 s and then rapidly and erratically increases for the last 100 s (grey filled symbols) (**E**) shows Mean ± SEM current–voltage relationship of H3 induced current exhibiting a non-specific conductance with a reversal potential close to 0 mV (*n* = 73, biological replicates. **F** Mean power spectral densities of diastolic *E*_m_ before (black) and after (red) exposure to 0.1 µg ml^−1^ H3. Each obtained from 30 diastolic resting *E*_m_ traces (0.6 s), zero-meaned, and windowed by a Hanning function, and its power spectrum computed using the Fast Fourier Transform in OriginPro, and the power spectra averaged to give 60 degrees of freedom spectral estimate for 0 < *f* < 5 kHz with a resolution of 5 Hz. The 95% confidence limits are presented (**G**) The difference between the two spectra of (**F**), i.e., the difference spectrum, is plotted and fitted by a Lorentzian function with a corner frequency of 3.21 ± 85.7 kHz.

The effect of A803467 and nifedipine on the peak amplitude of these currents along with representative traces are shown in Fig. 3A–C. A nonselective leak developed slowly for 300 s and then rapidly and erratically increases for the last 100 s (Fig. 3D) after H3 (0.1 µg ml^−1^) was added in the presence of both blockers within a mean time of 304.2 ± 56.7 s (*n* = 73). This value was not significantly different (*p* = 0.33) from the time for the *E*_m_ loss shown in Fig. 1J at the same concentration of H3. Control experiments that included both channel blockers but excluded H3 application did not develop a leak current over 500 s, only small fluctuations with a coefficient of variation of 6.8% (*N* = 167). The current voltage relationship shows the leak current resulting from H3 superfusion has a reversal potential of 0.13 ± 0.02 mV (Fig. 3E), and a specific conductance of 0.136 ± 0.074 mS/cm^2^ (*n* = 6).

Membrane potential changes produced by exposure to H3 during periodic pacing are followed for 500 s in Supplementary Fig. 2. By 300 s the triggered repetitive discharge is irregularly interspersed by depolarizations, initially of a few mV and with a half-width of ~ 1 s, these progressively increase in amplitude and duration until the *E*_m_ is lost at 550 s, a similar time course to that of the leak current under repetitive voltage clamp in Fig. 3D. The *E*_m_ changes smoothly throughout the recording, with ms to seconds kinetics, and APs continue to be evoked. Continued ionic channel functioning within a maintained cell membrane can be assumed, with *E*_m_ determined by electrodiffusion, pumps, and exchangers. For APs triggered within the slow, prolonged depolarizations shown in Fig. 1A–F and between 300 and 500 s of Supplementary Fig. 2, dV/dt_max_ was 80.96 ± 1.11 V s^−1^, showing a reduction of 37.4% from the control APs before H3 (129.39 ± 0.94 V s^–^¹). Assuming an *I*_Na_ reversal potential of approximately +60 mV, the depolarized *E*_m_ would reduce the electromotive force driving *I*_Na_ by ~30%, and since dV/dt_max_ is used as an indicator of maximal *I*_Na_, these results suggest that H3 exposure does not directly reduce fast Na⁺ conductance, and that the observed decrease in dV/dt_max_ is largely explained by the reduced driving force for Na⁺ entry. During 400 s of exposure to H3 (0.1 µg ml^−1^) the cell resting instantaneous input conductance estimated from the voltage deflection produced by the triggering pulses at the resting potential did not change significantly (0.030 ± 0.00026 before, 0.029 ± 0.0008 µS after the 400 s exposure.

Cells voltage-clamped at their resting *E*_m_ while exposed to H3 for up to 500 s exhibit erratically occurring ~100pA current pulses (Supplementary Fig. 3) with a peak amplitude, duration, and decay time constant that all increase with time.

In addition to depolarizations and changes in AP shapes, histones produce an increase in diastolic membrane voltage noise preceding each 1 Hz stimulus. This is visible in Fig. 1A–H and can be quantified by its standard deviation during the 600 ms preceding the next evoked AP. The standard deviation of these diastolic potential fluctuations increased from ±0.4 to ±2.28 mV (*n* = 5) following application of H3 at 0.1 µg ml^-1^. The power spectral densities of the diastolic potential before and after H3, illustrated in Fig. 3F, show an increase in the spectral density of 10^−3^ to 10^−2 ^mV/Hz at frequencies < 1 kHz produced by H3. Each spectral density is the mean of estimates from 30 non-overlapping 0.6 s blocks. The difference spectrum (Fig. 3G) has been fitted by a Lorentzian with a corner frequency of 3.2 kHz corresponding to a time constant of 0.31 ms, much faster than the slow increases in intermittent increases in intracellular Ca^2+^ (Fig. 2) or depolarizations (Fig. 1). The Lorentzian shape and corner frequency of the difference spectrum is dominated by the complex impedance of the membrane and the ten-fold increase in spectral density is consistent with the formation of large conductance pores.

### Histone released from necrotic cardiomyocytes caused E_m_ disruption with toxicity influenced by acetylation

**Fig. 4:**
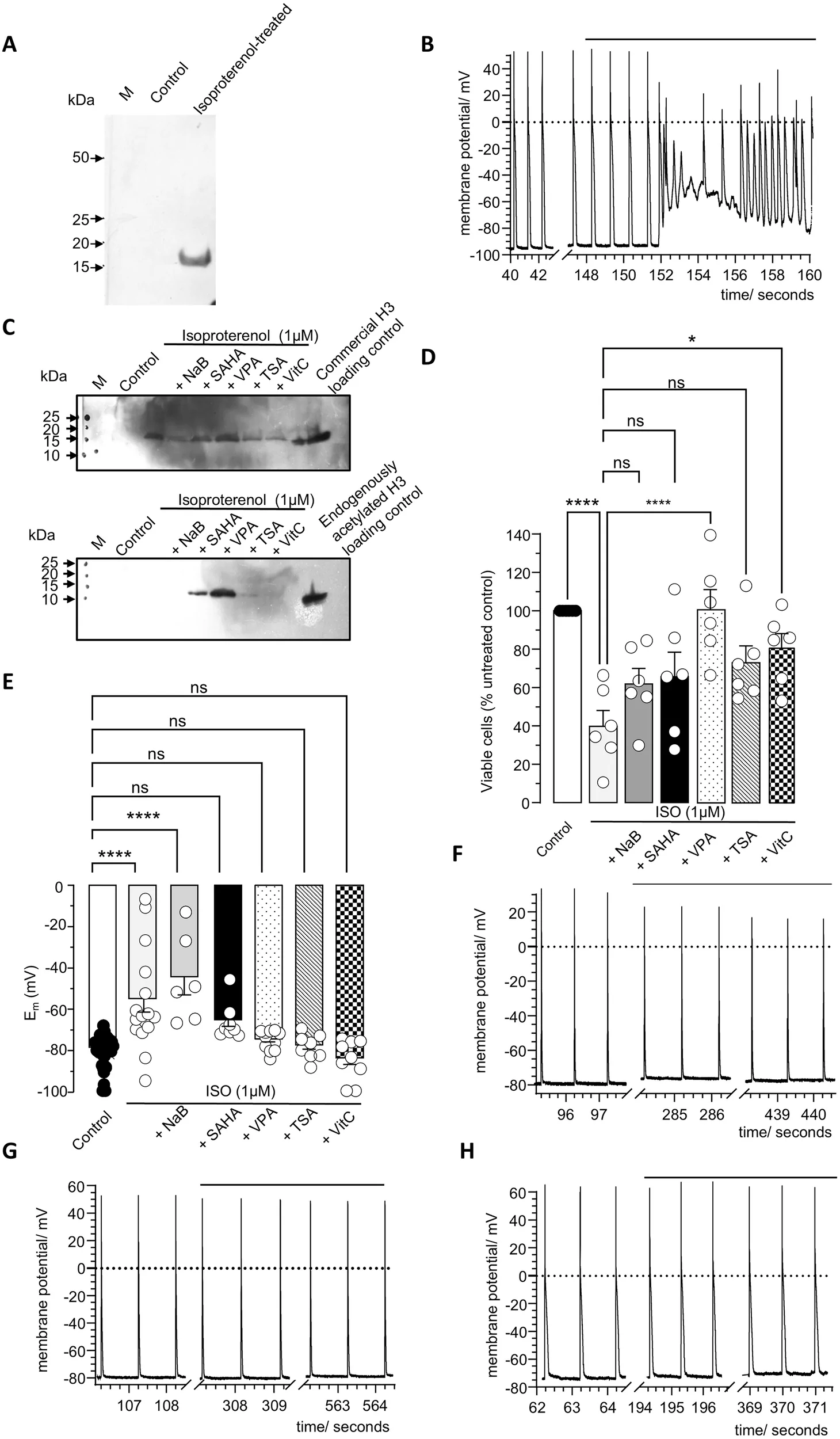
Media release of endogenous histone H3 from damaged cardiomyocytes and the influence of in vivo acetylation on toxicity. **A** Western blot image of locally produced endogenous H3 detect in the culture media after 24 h of isoproterenol treatment (1 µM) of cultured cardiomyocytes; untreated (Control). **B** Representative recordings of triggered APs before and during superfusion of isolated H3 from culture media (horizontal bar). **C** Western blot image of media H3 after 24 h without (control) and with isoproterenol treatment (1 µM; horizontal bar) and HDAC inhibitors as indicated. Precision Plus Protein Dual Color Standards (BioRad, Hertfordshire, UK) were used as a marker, labelled as ‘M’ in both (**A**, **C**). The expected molecular weight for H3 protein is 15 kDa. **D** Cardiomyocytes viability (mean ± SEM) for conditions shown, following isoproterenol treatment (1 µM; horizontal bar). **E** Mean ± SEM of measured *E*_m_ before (control; white) and after superfusion with media-released H3 isolated following 24 h of exposure of rat ventricular cardiomyocytes either alone or in combination with HDAC inhibitors agents. Each circular symbol represents individual data point; *P* values obtained from one way ANOVA with Bonferroni’s post hoc test, ***P < 0.001, ****P < 0.0001. Representative recordings of triggered APs before and during superfusion of isolated H3 from a 24 h cardiomyocytes culture media (horizontal bar) treated with combination of isoproterenol and TSA (**F**), isoproterenol and VPA (**G**), or isoproterenol and VitC (**H**).

To test if damaged cardiomyocytes membrane would result in histones being released outside the cells, and possibly cause further damage to healthy cells, isoproterenol (1 μM) was used to induce in vitro cell damage to cultured cardiomyocytes [29]. In 6 experiments, exposure to isoproterenol reduced cell viability by 60.1% ±11.7 compared to untreated controls, in close agreement to previously reported [30]. Histone H3 protein was detected after isoproterenol treatment by Western blots (*n* = 5) carried out on the culture media: a representative image shown in Fig. 4A (full length shown in Supplementary Fig. 4A).

The vehicle control, dimethyl sulfoxide (DMSO; 0.0002%), did not result in H3 being released in the cardiomyocytes culture media (Supplementary Fig. 4A) or significantly alter the cardiomyocytes resting E_m_ (Supplementary Fig. 4B, C).

To examine its toxicity, media H3 was purified and applied on freshly isolated cardiomyocytes during current clamp mode recordings. Figure 4B shows representative AP recordings before and during superfusion of media H3. Before H3 application, the diastolic *E*_m_ fluctuations were small with a mean ± SEM of 1.67 ± 0.2 mV (*N* = 40); following media H3 superfusion, there was a significant membrane depolarization shift and appearance of spontaneous electrical activity similar to that seen in Fig. 1. The resting *E*_m_ before media H3 application was −78.1 ± 1.96 and after exposure to media H3 of −55.98 ± 5.69 mV (*n* = 179; *p* = 0.00083). Overall, 64.7% of cells examined developed EAD-like oscillations, a pathological sign and so indicative of toxicity of the applied H3. This toxicity could result from high surface positive charge of H3 that could be damaging the cell membrane thus, neutralizing these charges may aid in reducing H3 toxicity. One possible approach to do this is *via* acetylation using histone deacetylase (HDAC) inhibitors; Sodium butyrate ([31]; NaB), Suberoylanilide hydroxamic ([32]; SAHA), Valproic acid ([33]; VPA), Trichostatin A ([34]; TSA), or using vitamin C ([35]; VitC) to influence the acetylation process [36]. Each drug was added in combination with isoproterenol, and cells were cultured as described above. Western blot analysis, carried out on the culture media, revealed that H3 was detected in all these examined conditions (Fig. 4C, upper image and Supplementary Fig. 5B). Moreover, acetylated H3 was detected in some of the conditions (Fig. 4C, lower image, and Supplementary Fig. 5B for full length image). The calculated % acetylation (band intensity of acetylated H3/ band intensity of total H3 ×100) can be found in Supplementary Fig. 3C. We have found that this approach was not quantitatively robust, as the acetyl antibodies varied in their detection capability. This could be due to the restricted acetyl modification groups for the antibody used (H3 acetyl K9, K14, K18, K23, K27); however, there was a significant improvement in the cell viability when compared to isoproterenol treated cells alone (Fig. 4D). Subsequently, media H3 was isolated from the acetylation conditions and its effects on *E*_m_ were examined. Figure 4E, shows summary data for *E*_m_ measured in all conditions; H3 isolated from isoproterenol treated media containing either VPA, TSA or VitC had a reduced effect on *E*_m_ and AP stability with the average duration of H3 superfusion of 379.3 ± 36.7 s before any APs changes, as illustrated Fig. 4F–H. EAD-like events were recorded in most cells examined, however, they appeared less frequently in cells recorded with H3 obtained from isoproterenol treated media containing either VPA or VitC (20% in each condition). Both VPA and VitC offer a partial protection against the myocyte membrane effects of H3, maybe due to an influence of the degree and/or sites of acetylation [32], and so may reduce its toxicity.

### Targeted In Vitro Acetylation of Histone H3 resulted in greater reduced toxicity

**Fig. 5:**
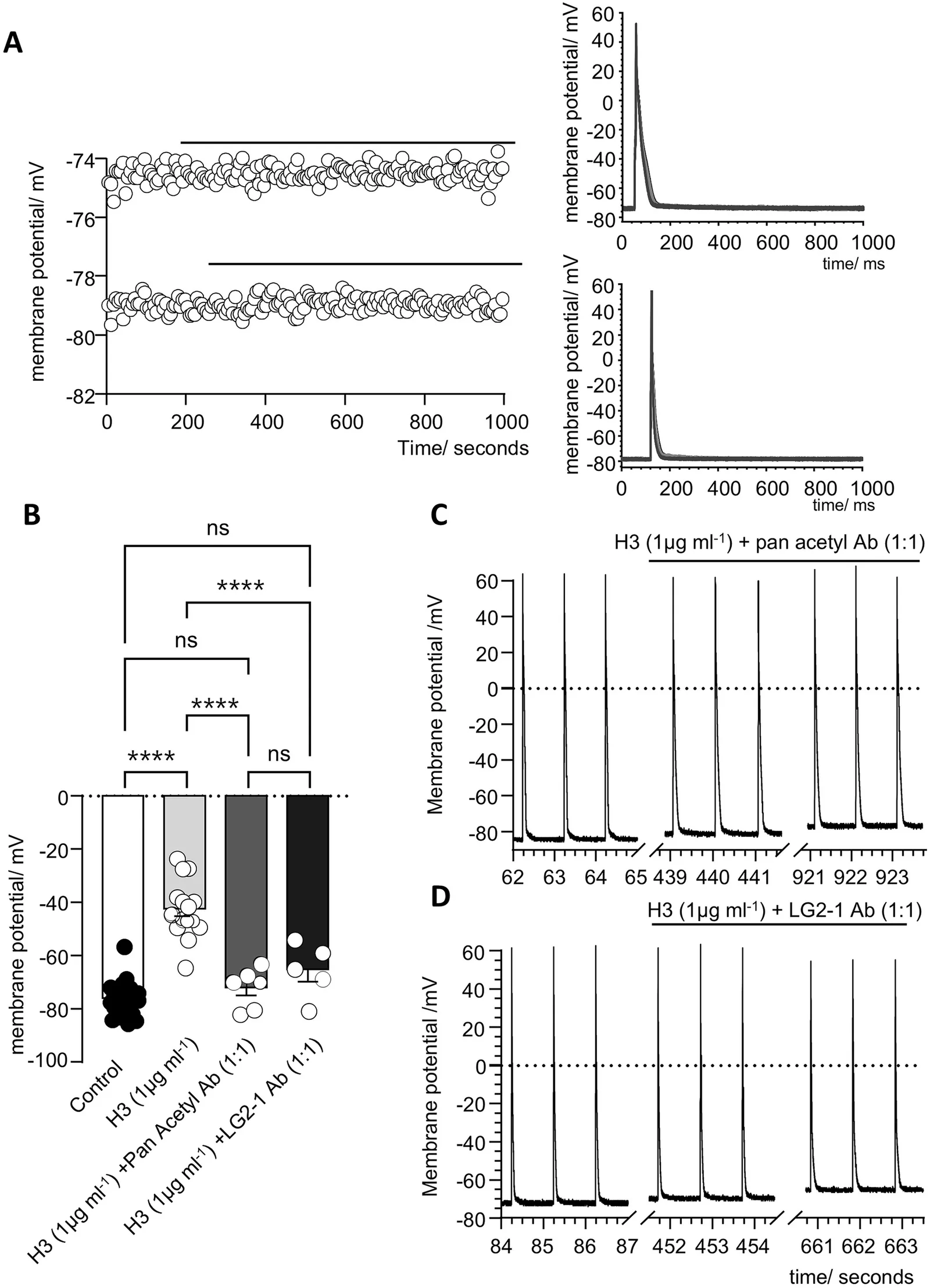
Targeted In Vitro Acetylation of Histone H3 reduces toxicity. **A** Time-series plots of averaged resting *E*_m_ for two ventricular myocytes (left panel) illustrating fluctuations before (control) and during superfusion of pan acetylated human recombinant H3 (activemotif, Waterloo, Belgium; horizontal bar) examined at concentration of 0.1 µg ml^−1^ (upper) or 1 µg ml^−1^ (lower) together their superimposed representative recordings triggered APs (right panel) before (black; *N* = 15) and during superfusion of pan acetylated H3 (grey; *N* = 15) (pan examined at 0.1 µg ml^−1^ (upper) or 1 µg ml^−1^ (lower). **B** Summary data Mean ± SEM of *E*_m_ measured at control condition (white) and after superfusion of commercially obtained H3 from calf thymus at a concentration of 1 µg ml^−1^ alone (grey) or as a complex with H3ac Ab° or as a complex with LG2-1 Ab° as indicated with **C**, **D** illustrating two representative recordings of triggered APs before and during H3 antibodies complex superfusion (horizontal bar) over examined time periods shown. Each circular symbol represents individual data point; *P* values obtained from one way ANOVA with Bonferroni’s post hoc test, ****P < 0.0001.

Further investigation into the role of acetylation in limiting H3 toxicity was assessed using synthetic pan-acetyl recombinant H3 protein (Activemotif, UK). Figure 5A shows examples of cardiomyocytes resting potential obtained after periodic (1/s) triggered APs recordings in control and during superfusion of pan-acetyl H3 protein applied at two different concentrations, along with superimposed triggered APs. Pan-acetyl H3 protein did not exhibit any significant effect on *E*_m_, resting potential remained constant with coefficient of variability in control condition of 0.125 and 0.068, while during Pan-acetyl H3 protein superfusion, resting *E*_m_ coefficient of variation was 0.057 and 0.031 measured for 0.1 µg ml^−1^ or 1 µg ml^−1^, respectively. Summary *E*_m_ value of −80.1 ± 1.38 for control and of −80.7 ± 1.9 during pan-acetyl H3 superfusion, with no spontaneous events observed up to 533.6 ± 146.4 s of recording (*n* = 94).

Additionally, antibodies (Ab°) were used to target charge neutralization of the commercial H3 preparation. Histone H3ac polyclonal Ab° (Invitrogen, UK) or anti-histone antibody LG2-1 [37] were incubated with H3 protein, the complex was applied during pacing at a concentration of 1 µg ml^−1^. The antibody-histone complex had a smaller effect on cardiomyocytes *E*_m_ compared to H3 alone (*n* = 159), where *E*_m_ disruption occurred within 127.1 ± 27.5 s as illustrated in Fig. 1I, J. Figure 5B shows summary data of these effects taken at 360 s, with *E*_m_ showing no significant change before and during the superfusion of H3 and Histone H3ac Ab complex (6 out of 7), or H3 and LG2-1 antibody complex (5 out 7). In some cells recording up to 600 s were obtained showing little change when compared to controls as illustrated in the representative traces in Fig. 5C, D. Overall, the occurrence of spontaneous events was reduced by charge neutralization. 78.5% of the cells developed histone evoked depolarizations and bursts following exposure to 1 µg ml^−1^ concentration of commercial H3 without any charge neutralization. However, when charge was neutralized using either of Histone H3ac or LG2-1 antibodies, only 42.8% and 28.5% of cells exhibited EAD-like events respectively.

## Discussion

This study demonstrated that low levels of histones (ng to μg ml^−1^) can in vitro induce *E*_m_ depolarizations, SR Ca^2+^ dysfunction, prolongation of APs, and EAD like events in isolated cardiomyocytes.

In clinical settings, levels of histones at ≥ 75 μg ml^−1^ do occur in sepsis patients with an associated significant poor survival rate [16]. Circulating histones levels were also detected by mass spectrometry as a diagnosis tool developed for septic patients’ management [38] with levels as low as 0.12 µg ml^−1^ in patients with severe septic shock. Levels above 0.43 µg ml^−1^ were associated with organ failure, however, the histones levels within intra-cardiac tissue during severe sepsis are not known precisely, but are likely to be in or above the range that causes membrane damage, as rising levels of histones in sepsis correlate with the appearance of cardiac-specific troponin in blood [13,14], an index of cardiomyocyte death, and arrhythmia detected in over 50% of mice infused with 50 mg/kg histones [39]. Additionally, fluorescent histones could be detected within 30 min in murine myocardial tissues isolated from mice that were previously intravenously injected with 45 mg/kg body weight fluorescent histones [16] and increasing histone levels observed after the induction of myocardial infarcts by coronary artery ligation [40]. In Langendorf-perfused rat hearts myocardial ischaemia-reperfusion injury produced the release of up to 30 µg ml^−1^ H3, and the histone release and toxicity were not reduced by inhibition of TLRs, activation of inflammatory pathways [41].

Thus, in clinical sepsis, myocardial cells are likely to be exposed to levels of histones that can cause direct membrane damage, and acute membrane disruption is likely to be a major contributor to myocardial dysfunction in severe sepsis. In contrast, situations in which exposure of cardiac cells to histones over a much lower concentration range, and/or where there is a gradual increase exposure to histones, have been reported clinically, for example, a slow rise in plasma histone levels to 20 μg ml^−1^ can be detected following myocardial infarction [40–42]. Moreover, following physical trauma histones released into circulation are related to the injury level; a lower level of trauma will result in lower levels of circulating histones [7]. Cells can be exposed to low (ng ml^−1^ range) and prolonged or gradually increasing level of histones.

Ventricular fibrillation, the last wave of the dying heart, precedes or accompanies virtually all deaths, and although it is the manifestation of irregular interacting propagation in the myocardium that leads to circulatory collapse, it can be understood in terms of the electrophysiology of isolated cardiomyocytes. Action potential prolongation and disorders in repolarization can trigger arrhythmia and maintained cell depolarization can lead to conduction blocks and breakdown of normal sinus rhythm into spatiotemporal irregularity [43] and provide quantitative measures of both the intensity and time scale of processes leading to the death of cells and the probability of death of whole individual. This focus on cardiomyocyte to cardiac death neglects the role of interactions within the extracellular fluid (histones bound to DNA in (mononucleosomes), plasma, tissues (interactions with vascular epithelial cells amplifying inflammatory responses, neutrophil uptake and release as neutrophil extracellular traps as an anti-microbial response, and organ systems, that are all simplified to the resultant extracellular concentrations of free histones.

Histones (0.01–1 µg ml^−1^) consistently perturbed the activity of paced isolated ventricular cardiomyocytes within a few hundred seconds, acting on the *E*_m_ and producing action potential and intracellular Ca^2+^ dysfunction that would be pro-arrhythmogenic in ventricular myocardial tissue and potentially fatal in vivo. Both the *E*_m_ and intracellular Ca^2+^ activity indices change erratically but progressively with time, with the depolarizations and Ca^2+^ events progressively increasing both in magnitude and duration time.

Isolated cardiomyocytes are ~100 × 30 µm cylinders and so are effectively isopotential, while intracellular Ca^2+^ is sequestered into organelles (sarcoplasmic reticulum, mitochondria) and bound to any accessible negative charges, mostly of proteins. The bulk (50–60% by dry weight) of the intracellular proteins are spatially organized into architecture of the sarcomere. Intracellular Ca^2+^ is spatially organized and can be mapped in 3D by confocal microscopy using calcium sensitive dyes and fluorophores [44], with localized sparks, propagating waves, tides, and global oscillations of Ca^2+^ activity. Computational models of myocyte electrophysiology [45,46] couple sarcolemmal fluxes of Ca^2+^ to four intracellular compartments (bulk myoplasm including mitochondria, junctional sarcoplasmic reticulum, network sarcoplasmic reticulum, and subspace near the transverse tubules. The congruence of the Ca^2+^ line scan images and their whole cell average *F*/*F*_0_ time series of Fig. 2 show that over 1 ms to 10 s time scales the Ca^2+^ transients were driven by cell level rather than intracellular wave processes.

Histone-induced changes in paced and spontaneous Ca^2+^ transients could reflect increased Ca^2+^ influx or increased release from intracellular sites. During an AP, Ca^2+^ entry through L-type Ca^2+^ channels into the subcellular space triggers Ca^2+^ release from the sarcoplasmic reticulum, and Ca^2+^ rises to a few hundred nM [46–49] and falls over several hundred ms as it is pumped back into the sarcoplasmic reticulum and exchanged by the sodium-calcium exchanger (NCX) with a time scale of seconds. Any sustained increases in mean [Ca^2+^]_i_ levels require Ca^2+^ influx in cardiomyocytes. The peaks of the triggered Ca^2+^ transients are reduced by H3 and H4, and the irregular Ca^2+^ oscillations are around a level about a third of the peaks of triggered control responses. Both the *E*_m_ and intracellular Ca^2+^ activity indices change erratically but progressively with time, with the plateau depolarizations and Ca^2+^ events progressively increasing both in magnitude and duration time. Either there is a >100 s time course or an accumulatory process.

In the voltage clamp experiments of Fig. 3A–E, NCX activity was blocked due to the use of calcium chelator in the intracellular solution. Following H3 application, a non-specific leak with a linear current-voltage relation (Fig. 3E) was consistently developed, providing an indication by another route for the histone-induced Ca^2+^ influx.

If this histone-produced leakage conductance was the result of inserting unitary conductance channels in the membrane, the total number N of unitary conductance channels/unit area of the membrane would increase, and the coefficient of variation of the membrane noise would scale as 1/N^2^, i.e decrease. Figure 1A–F illustrate a small increase in resting *E*_m_ noise, and Fig. 3F an increase in *E*_m_ spectral density, implying suggesting the incorporation of a small number of events with a large effect, rather than a large number of events each with a small effect.

The Lorentzian spectrum of the noise induced by H3 is consistent with a rapidly activating channel or a rapid induction of a nonspecific depolarizing current with a *linear* current voltage relationship and a reversal potential of ~0 mV, as through large pores. From Fig. 3E, the conductance produced by 0.1 µg ml^−1^ H3 is 18.5 mS/µF, larger than the ion specific maximal conductances of rat and human of cardiac myocyte voltage dependent channel systems. We interpret the data in Figs. 1A, 3F and Supplementary Figs. 2 and 3 as suggesting large and variable-sized pores in the membrane, rather than membrane lipid disorganization.

Early reports describing histone interaction with lipid and phospholipids bilayers and liposomes implied concluded that histones may form pores of varying sizes [50–52]. Technological limitations unable us to investigate this mechanism further, a combination of single channel patch clamp, voltage clamp noise membrane current analysis, atomic force microscopy, and conductance microscopy would be required to investigate this in isolated cells. Ion conductance microscopy has visualized ~0.5 µm diameter pores following H4 on the surface membrane of vascular smooth muscle cells [53], three orders of magnitude larger than that of excitable membrane ionic channel.

An increase in extracellular histone concentration or increased binding of histones to molecules on the membrane surface could induce pore structures that could disrupt the electrochemical gradient and lead to cell death [54]. Fluorescein labelled histones can enter cardiomyocytes [11,16], perhaps through this pathway, and may also have further deleterious intracellular effects.

A well characterized example of such a process is the transmembrane toxin colicins produced by *E. coli*, that contain regions with a high concentration of positively charged amino acids and lead to depolarization of the cytoplasmic membrane and pore formation *via* electrostatic interaction [55]. This results in ion leaks, depolarization, reduced electrochemical gradient, and subsequently reduced ATP availability and cell death [56,57]. Our evidence suggests that similar non-specific membrane damage following exposure to histones that lead to depolarization *via* a non-specific conductance, and an increase in Ca^2+^ influx [58]. Histones have been shown to induce membrane leakage [54], inducing inward ion current [7], interacting and integrating directly with membrane phospholipids [54,59].

We also demonstrated a cytotoxic effect of H3 produced locally following cardiomyocyte injury, providing evidence that histones released endogenously can further damage adjacent healthy myocardial cells, possibly spreading slowly through tissue as a wave of induced histone release and diffusion. Histone release into the surrounding media has been recently shown to be associated with cellular stress and mediated by extracellular vesicles [59], and extracellular histone binds to lipid membranes of vesicles, cells, and lipid bilayers. A localized or a spreading tissue injury due to histones release could result in increase of developing cardiac pathologies, including arrhythmia, heart failure, and sudden cardiac death [15,60]. A rising level of histone H4 has been detected in the perfusate of rats’ hearts following ischemia/reperfusion injury [41]. Other studies conducted on pancreatic mouse cell lines and animal models showed that inducing necrosis resulted in the release of histones in either bound or unbound forms; these released histones lead to biomarkers associated with damage patterns and/or further tissue and organ damage [61,62].

Action potential prolongation and disorders in repolarization can trigger arrhythmia, and maintained cell depolarization can lead to conduction blocks and breakdown of normal sinus rhythm into spatiotemporal irregularity [43,63] of ventricular fibrillation. The last waves of the dying heart precede or accompanies virtually all deaths. Although it is the manifestation of irregular interacting propagation in the myocardium that leads to circulatory collapse, it can be understood in terms of the electrophysiology of isolated cardiomyocytes.

The actions of histones on cardiac electrophysiology provide quantitative measures of both the intensity and time scale of processes leading to the dysfunction of myocytes and the resultant probability of death of whole individual.

There is a growing support indicating evidence that extracellular histones are toxic mediators in pathologies involving cell injury, inflammation, and reperfusion damage. In a sepsis mouse model, a significant increase in circulating cardiac troponin T level was produced within 6 h when circulating H4 reached a plasma level of ~250 pg ml^−1^ [14]. The low levels of local histone concentrations produced during sepsis can impact the myocardium and be detrimental. However, histone toxicity can manifest differently between rodents (rats/mice) and humans in the sensitivity of examined cells. These translation aspects should always be considered when determining toxicity levels for pre-clinical studies. For example, when H4 was examined on human neutrophils [64] at a high level of 40 µg ml^−1^ it directly stimulates activation through membrane permeabilization, Ca^2+^ influx, membrane depolarisation, and cytokine release (TNF-α, IL-1β). Concentrations between 32 and 256 µg ml^−1^ caused cell membrane obliteration of cell of HUVEC, HAEC, COS7, and HepG2 mammalian cell lines, as shown by scanning electron microscopy [65]. However, at lower levels of circulating histone (2.6–29.6 µg ml^−1^), clinical outcomes correlated with the concentrations [4,66]. Here, a concentration of 0.1 µg ml^−1^ showed toxic effects similar to these seen in rat cardiomyocytes (Fig. 1) when examined on human iPSC-derived cardiomyocytes (Supplementary Fig. 7). Our data demonstrate the intrinsic susceptibility of cardiomyocytes to histone toxicity; further in vivo studies will be essential to determine whether similar mechanisms operate under pathological conditions.

Previous studies have suggested that TLRs may have a role in histone-induced cardiac toxicity [67], and TLR2 & 4 are known to be present in myocytes [68]. However, our results show using TLR2 and TLR4 inhibitors fail to show a TLR role in the histone-induced cardiomyocytes toxicity (Supplementary Fig. 6). A similar independence of histone toxicity from TLR signalling of has been reported using human cell lines; HEK293 reporter cells expressing a TLR4 reporter [41] and human umbilical vein endothelial cells [69].

The histone shape/structure may also be important in contributing to its binding affinity to the anionic phospholipids [70]. Our work suggests that cardiomyocyte membrane disruption by histones is closely related to the positive charge of H3. The cytotoxic effect of H3 is reduced and eliminated when the synthetic acetylated version was examined, and by charge neutralization with antibodies. Moreover, acetylation *via* the use of selected HDAC inhibitors will reduce the positive charge and has led to improvements in cardiomyocyte viability and a reduced cytotoxic response to locally produced H3 (Fig. 4). The acetylation process will reduce the positive charge. However, lysine acetylation is a reversible process [71], time- and residue-specific [32], which may explain the variability with some of HDAC inhibitors used here. Inhibition of HDAC has been shown to reduce ventricular remodelling in a mouse model of transverse aortic constriction, resulting in enhanced cardiac function [72].

Neutralization of lysine charge via methylation may play a role in enhancing these charge changes [73]. VitC has been shown to lead to enhanced H3 Lysine-4 methylation [74], while VPA increases H3 acetylation levels [75] and modulates lysine residue methylation [76]. Both VitC and VPA offered over 80% protection against the development of EAD-like events (Fig. 4). A general charge neutralization approach using heparin [77,78] as a pharmacological agent was limited (Supplementary Fig. 6). Although heparin contributes to the management of sepsis-associated complications by reducing coagulation dysfunction, it has not been associated with a reduction in time to death [79]. In contrast, selectively desulfated heparin, characterized by diminished anticoagulant activity, has demonstrated substantial efficacy as an anti-histone agent [80].

A more specific targeted charge neutralization approach seems more appropriate, for example, using the LG2-1 antibody that contains large numbers of negatively charged amino acid residues [37], designed to react with residues 30–45 of H3. These residues include two Lysine residues at positions 37 and 38, both predicted sites for histone acetylation and methylation, respectively, led to a similar degree of protection against the development of EAD-like events (Fig. 5).

In summary, histones are released from damaged cells in different tissues, as a result of oxidative stress, inflammation, sepsis and trauma, and lead to clinical levels of ng-100 µg that in vitro have pro-arrhythmogenic effects on cardiomyocytes that in vivo could trigger ventricular fibrillation and death. Neutralization of the positive charged groups on the histone molecules reduces the electrophysiological effects on cardiomyocytes and increases favourable outcomes in vivo and clinically. Polyanionic peptides designed to optimize binding to the positive sites of H3 and H4 could reduce the spread of histone-induced cell damage and increase survival rates following large-scale tissue damage.

## Methods

### Isolation of ventricular myocytes

Cardiomyocytes were isolated from adult (approximately 3 months old) male Wistar rats (150–200 g) ventricles as described [81], obtained from 79 preparations, in accordance with UK Home Office Guidance on the Operation of Animals (Scientific Procedures) Act 1986; the project used a UK Home Office Schedule 1 procedure only. The project was approved by the Animal Welfare and Ethical Review Committee of the University of Leeds (Approval number AWCNRWDS130706) and Institutional guidelines. Briefly, Langendorff mounted hearts were perfused with prewarmed (37 °C), oxygenated isolation solution (in mM; NaCl 130, KCl 5.4, MgCl_2_ 1.4, NaH_2_PO4 0.4, HEPES 5, glucose 10, taurine 20, creatine 10, pH 7.3) supplemented with CaCl_2_ (750 μM) for 3–4 min to remove blood from heart cavity, followed by Ca^2+^ free isolation solution containing EGTA (100 µM) to stop the heart beating, subsequently Ca^2+^ free isolation solution containing collagenase (Worthington type 2; 1 mg ml^−1^) and protease (0.1 mg ml^−1^) were used for the digestion process for 9–12 min. The ventricles tissues were removed, cut into smaller pieces in the enzyme containing solution with added bovine serum albumin (1%), and agitated in a water bath at 37 °C. Cell suspensions were examined every 5 min until rod shaped cells with a clear striation pattern were obtained by filtration. Cells were gently centrifuged, then washed and stored in isolation solution containing CaCl_2_ (750 μM).

### Electrophysiology

Whole cell voltage-clamp recordings were obtained as previously described [82,83], myocytes were placed in recording chamber on the stage of an inverted microscope (Olympus CK40; Olympus, London, UK) for 10-15 min to settle before superfusion with extracellular solution (in mM: 140 NaCl, 4 KCl, 1.8 CaCl_2_, 1 MgCl2, 5 glucose, 10 HEPES, pH 7.4). Patch electrode resistance was between 4 and 6 MΩ when filled with intracellular solution (in mM: KCl 130, NaCl 10, EGTA 5, HEPES 5, MgATP 5, CaCl_2_ 1, MgCl_2_ 1; pH 7.2). In current clamp mode recorded action potentials (AP) were triggered every 1 s by current injections of 0.4 nA and 5 ms duration. Action potential durations (APD) were measured at 50 (APD_0.5_) and 90% (APD_0.9_) of repolarization. Ionic currents (*I*_Na_ and *I*_Ca_) recordings were made in voltage clamp mode using a linear voltage ramp from –100 to +60 mV over a 500 ms period, with pre step from −40 mV to −100 mV for 50 ms, applied every 3 s. Myocytes were perfused with a solution containing (in mM: 140 NaCl, 5.4 CsCl, 1 MgCl_2_, 2 CaCl_2_, 10 HEPES, 5 glucose, 20 TEA-Cl, pH 7.4 with NaOH). Patch electrodes were filled with a solution containing (in mM: 120 CsCl, 20 TEA-Cl, 2 MgCl_2_, 10 EGTA, 10 HEPES, pH 7.2 with CsOH). A803467 [26] and Nifedipine [28] were applied to perfusate to block *I*_Na_ and *I*_Ca_, respectively, before the addition of H3 (0.1 µg ml^−1^). Whole cell configuration, for voltage or current clamp experiments, was recorded, digitized and stored with an Axopatch 200B amplifier, Digidata 1550B and pCLAMP 11 respectively (Molecular Devices, USA). The integrity of the high GΩ resistance seal was monitored during experiments, and the experiment was terminated if more than a 20% increase in series resistance occurred’.

### Histone proteins and HDAC inhibitors stock preparation

Stocks of commercial preparations of histone proteins; H3 isolated from calf thymus (Sigma–Aldrich, UK), human recombinant H4 (New England Biolabs, UK) and synthetic pan-acetyl recombinant H3 protein (Activemotif, UK) were prepared in advance in phosphate buffer solution, aliquoted, and stored at −80 °C until needed. On the day of experiments, stock aliquots were kept at 4 °C and diluted further in extracellular solution to achieve the desired concentration. Valproic acid (4 M), sodium butyrate (100 mM) and vitamin C (200 mM) stocks were prepared in distilled deionised water, TSA (5 mM) and SAHA (5 mM) were prepared in DMSO.

### Confocal imaging of intact myocytes

Confocal imaging was obtained using a laser-scanning unit (BioRad Microradiance 2000, UK) attached to a Nikon Diaphot inverted microscope (Nikon; Tokyo; Japan) as previously described [79,80]. Briefly, intracellular calcium levels were measured following loading myocytes with the fluorescent Ca^2+^ indicator Fluo-4 (6 μM; Thermo Fisher Scientific, UK) for 15 min at room temperature. Myocytes were washed and resuspended in Tyrode solution with CaCl_2_ (1 mM) and stored for at least 30 min at room temperature to allow de-esterification of the dye. Fluo-4 was excited at 488 nm and emitted fluorescence measured at >515. Grass S48 stimulator was used to field stimulate myocytes with pulses delivered via a platinum electrode (0.2 mm diameter; Goodfellow Metals) positioned alongside the low volume cell chamber. Voltage pulses (6 ms duration wave, 0.5 Hz) were applied at 1.5 times the threshold voltage for an electrically evoked contraction. To enhance reproducibility, myocytes that failed to respond to electrical stimulation or showed signs of spontaneous activity under control conditions were excluded (*n* = 6, for both H3 and H4 experiments). Images were analysed using ImageJ (NIH, USA, http://rsbweb.nih.gov/ij/).

### Culture of adult cardiomyocytes and treatments

Cardiomyocyte necrosis was induced by incubation of the cells with the β-adrenergic agonist isoproterenol (ISO; 1 µM) for 24 h under cell culture conditions using Medium 199 in six well plates. Myocytes were incubated at 37 °C in a humidified incubator with atmosphere of 5% CO_2_. Histone deacetylase (HDAC)-mediated deacetylation inhibitors: NaB (0.5 mM), SAHA (1 µM), VPA (4 mM), TSA (1 µM), or VitC (200 mM) were added during isoproterenol treatments (total media volume = 2 ml); the final concentration of DMSO did not exceed 0.0002%. Untreated cells were used as a control. After 24 h incubation, the media from cultured myocytes with or without drugs were collected and centrifuged to remove any debris. To ensure removal of treatment drugs and solvents present in the media before obtaining any electrophysiological recording, the supernatant was filtered, buffer exchanged using an Amicon Pro purification system as instructed by the manufacturer (Sigma–Aldrich, UK). A BCA assay yielded a concentration between 0.1 and 0.7 µg ml^−1^. Cell viability was assessed using haemocytometer; viable cardiomyocytes appeared rod-shaped with a clear striation pattern and sharp edge; cardiomyocytes with diffuse outer membrane or rounded were identified as nonviable.

### Immunoblotting

Media samples taken after myocytes culture were subjected to electrophoresis on 15% precast mini- PROTEAN gels, then transferred to PVDF membranes according to the manufacturer’s protocols (Bio Rad, UK). Unless otherwise stated, all steps were performed at room temperature and subjected to washes in Tris Buffered Saline (TBS; Bio Rad, UK) containing Tween 20 (TBST; 0.05%; 3 × 5 min) between each step. Membranes were blocked using 5% non-fat milk protein in TBST for 1 h, immunostained with histone H3 antibody (Santa Cruz Biotechnology, UK) or anti-Histone H3 acetyl K9 + K14 + K18 + K23 + K27 antibody (Abcam, Cambridge, uk) prepared in 1% non-fat milk protein in TBST for 1 h, and then incubated with secondary anti-mouse Ig antibody conjugated to horseradish peroxidase (Santa Cruz Biotechnology, UK) for 1 h. Immunoreactive bands visualized using the enhanced chemiluminescence (ECL) detection system (LI-COR Biosciences, UK).

### In Vitro formation of Histone Protein H3: antibody complex

Histone H3ac polyclonal Ab° (Invitrogen, UK) and anti-histone antibody LG2-1, commercially produced and purified (BioServUK Antibody Production) from Mouse hybridoma cell lines producing anti-histone IgG’ Hybridoma cells (Kind gift from Professor Mark Shlomchik, Department of Immunology, University of Pittsburgh) were incubated with commercial H3, isolated from calf thymus (1 mg ml^−1^; Sigma–Aldrich, UK) at 1:1 ratio. Mixtures were placed on a rotating surface and incubated for 2–4 h at 4 °C and then diluted further in extracellular solution (in mM: 140 NaCl, 4 KCl, 1.8 CaCl_2_, 1 MgCl_2_, 5 glucose, 10 HEPES, pH 7.4) to be used for electrophysiological recording at a final concentration of 1 µg ml^−1^.

### Statistical information

Data from multiple independent experiments were pooled and analysed using Excel (Microsoft, UK) and Origin (Northampton, MA) software and presented as mean ± SEM. Biological replicates, a minimum of ≥3 hearts/animals were used for each experiment (Supplementary Tables 1). Histograms of the values of these variables, from repeated evoked activity in a single cell, or from the population of isolated cells, under the same conditions, were unimodal and approximately symmetric, and following the Shapiro–Wilk normality test it was concluded to be a Gaussian distributed. For statistical comparisons, data were assessed by Student’s *t*-test or one-way ANOVA with Bonferroni’s multiple-comparison test. A value of *p* < 0.05 was taken as statistically significant; “*n*” indicates the number of cells, followed by the number of animals/hearts in parenthesis; details can be found in Supplementary Tables. 1. “*N*” is the number of events.

## Supplementary information

Supplementary Information

Fig 4A - Original Data

Fig. 4C - Original Data

Suppl.jpg

## Data Availability

The data sets supporting the conclusions of this article are included within the article; any additional information is available on request from the corresponding author, m.al-owais@leeds.ac.uk.
