# Supplementary material for: Mechanisms of cardiomyocyte dysfunction induced by extracellular histones
Source: Cell Death Discov. 2026 Jul 27;12:422. doi: 10.1038/s41420-026-03274-w (PMC13690737; doi:10.1038/s41420-026-03274-w)
Supplement: Supplementary file 1 [file 41420_2026_3274_MOESM1_ESM.pdf]

Mechanisms of cardiomyocyte dysfunction induced by extracellular histones

Moza M. Al-Owais*[1], Zhaokang Yang[1], Derek S. Steele[1], Arun V. Holden[1]

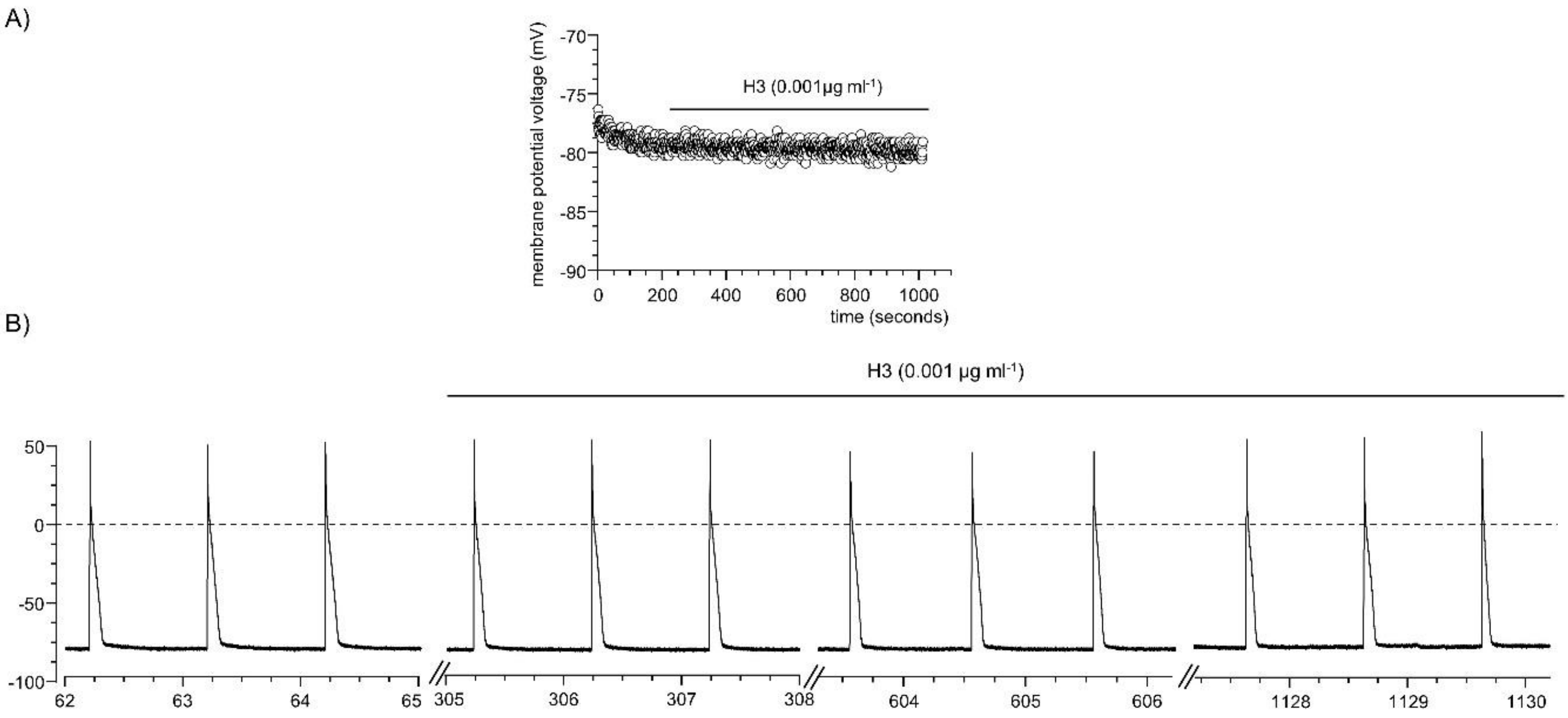

Supplementary Fig. 1. Stability of recording (A) Representative time series example illustrating the stability of diastolic membrane potential (mV) recording in current-clamp mode, over >1000s. The resting membrane potential was recorded during pacing by a 0.4nA, 5ms suprathreshold pulse train triggering an AP every second. The horizontal line indicates the period of H3 superfusion (0.001µg ml^{-1}). **(B)** Representative trace examples of the trigged AP and baseline voltage (mV) of the cell shown in A.

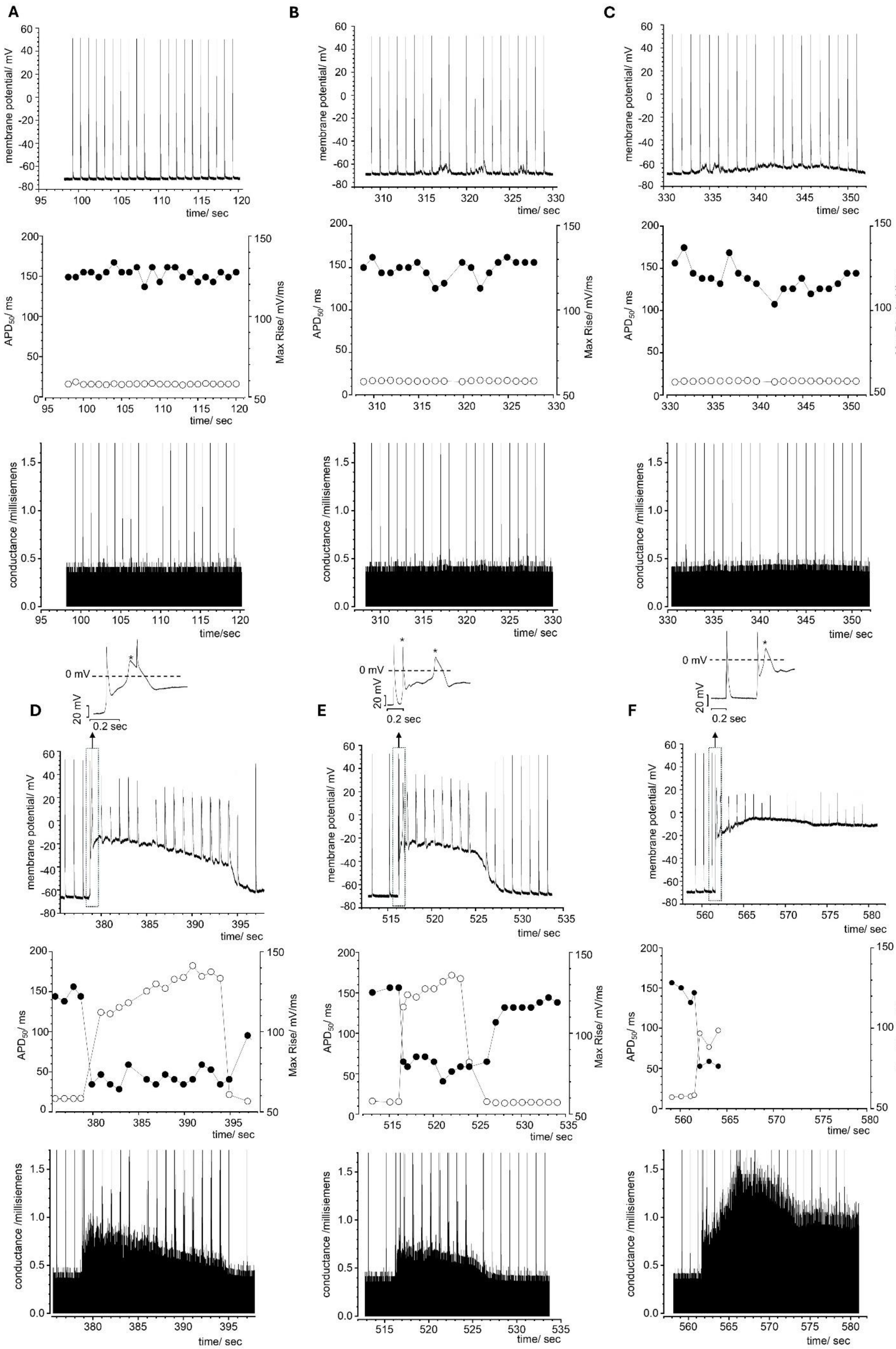

A
B
C
D
E
F
membrane potential/ mV
time/ sec
APD50/ ms
Max Rise/ mV/ms
conductance /millisiemens
time/sec
0 mV
20 mV
0.2 sec
*

Supplementary Fig. 2. Successive 20s blocks of membrane potential changes during 1 second pacing and exposure to H3 (0.1µg ml^{-1}). Pacing is by a train of 10 current pulses (5 ms, 0.4nA) every 11s. Control; (A) 100-120, H3 exposure selected periods; (B) 310-330, (C) 330-350, (D) 375-395, (E) 515-535 and (F) 560-580 s. Each panel displays voltage (top), APD_{90} and dV/dt_{max} (middle) of triggered and untriggered action potentials, and the specific membrane conductance mS/cm^2 estimated from $-(dV/dt_{max})/V$ (lower), assuming a specific membrane capacitance of 1 $\mu F/cm^2$ and a reversal potential of 0 mV. The triggered APs are clearly distinguishable from the trigger stimulus artefact; see zoom panels from D, E and F, membrane potential, with untriggered APs marked by *.

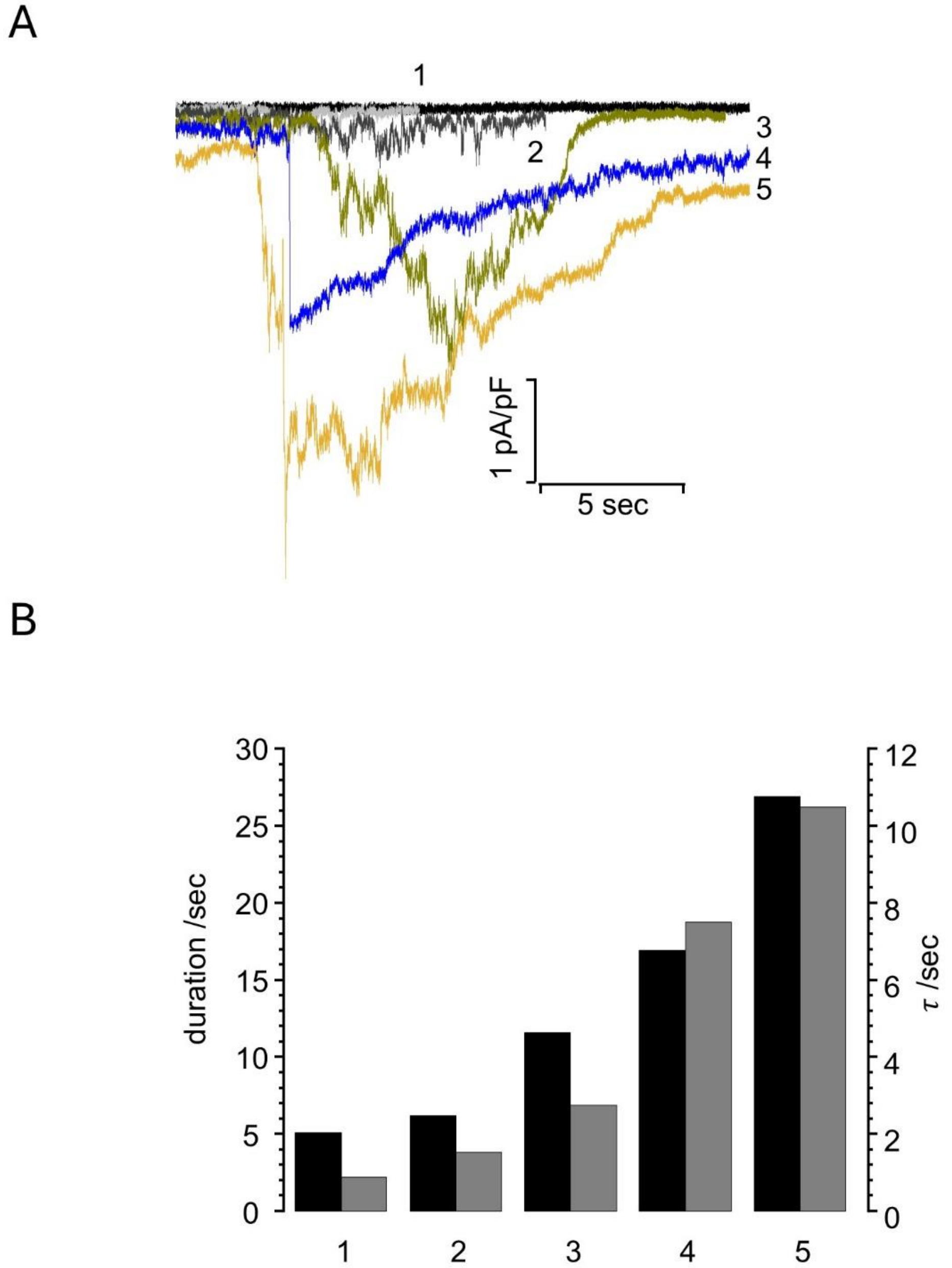

Supplementary Fig. 3 Spontaneous current transients during H3 exposure (0.1 μg ml^{-1}) during voltage clamp at cell resting membrane potential. (A) superimposed transients occurring progressively from time 130 seconds (1) to 300 seconds (5) times. (B) Progressive increase in duration and fitted exponential decay time constants for 1 to 5.

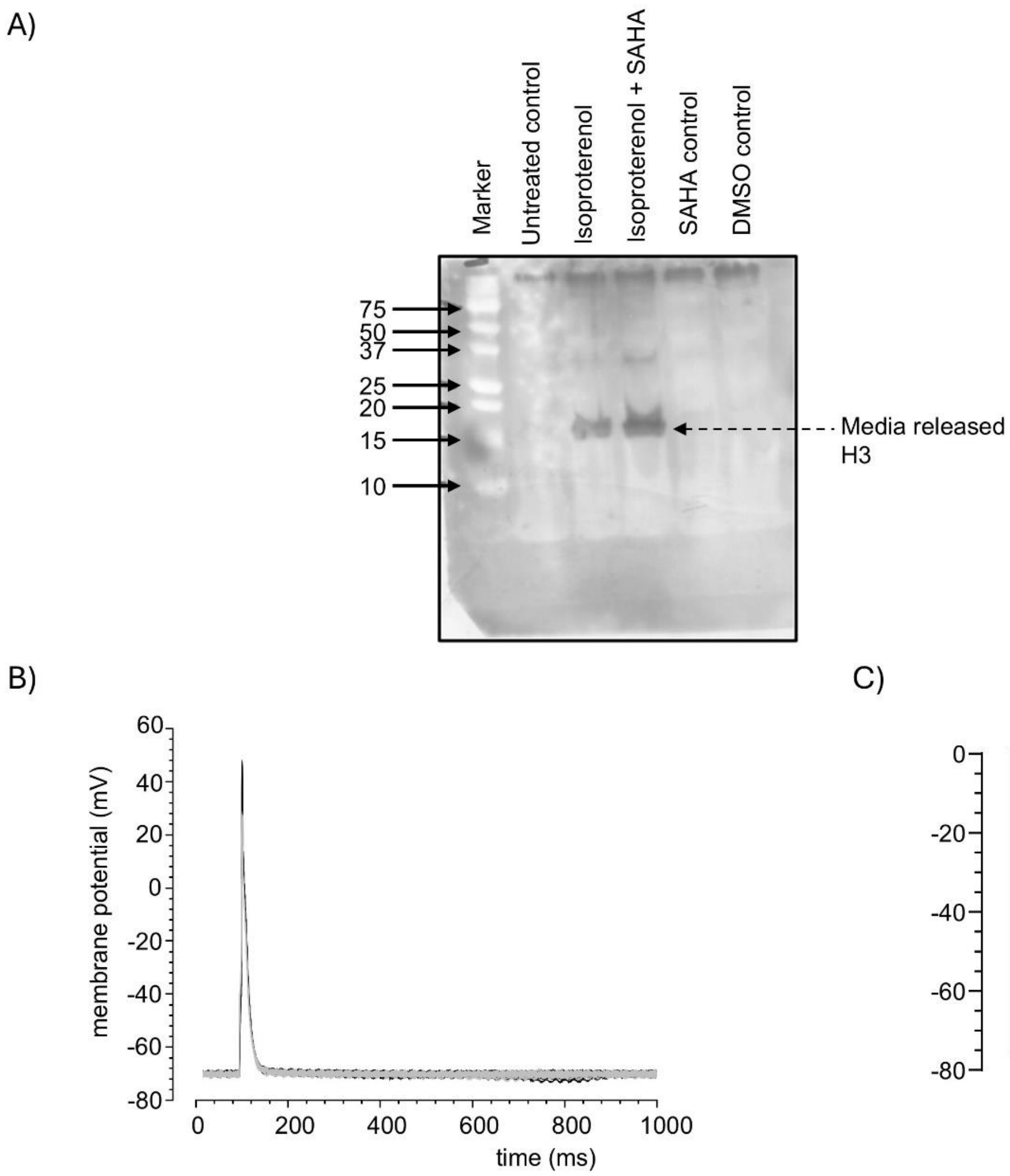

Supplementary Fig. 4. Controls (A) Western blot Image for samples obtained from cultured cardiomyocytes media following 24 hrs incubation with isoproterenol, isoproterenol and HDAC inhibitor suberoylanilide hydroxamic acid (SAHA). Experimental controls untreated (to rule out the H3 release), SAHA treated (to rule out a possible drug effect) and dimethyl sulfoxide (DMSO, 0.0002%) treated groups (to rule out a possible solvent effect). Samples were collected, ran on SDS-gel and transferred to PVDF membrane and H3 antibody was used to detected media released H3. H3 can only be detected in isoproterenol, and isoproterenol and SAHA conditions. All control groups were confirmed to be negative for H3 release. (B) Representative traces example during an action potential experiment before (black, N=20) and during superfusion of 0.0002% DMSO (grey, N=20) examined in rat ventricular cardiomyocytes. (C) Diastolic membrane potential (Mean ±SEM) recording obtained from a duration of 3 min, before (black, N=100) and during superfusion of 0.0002% DMSO (grey, N=100).

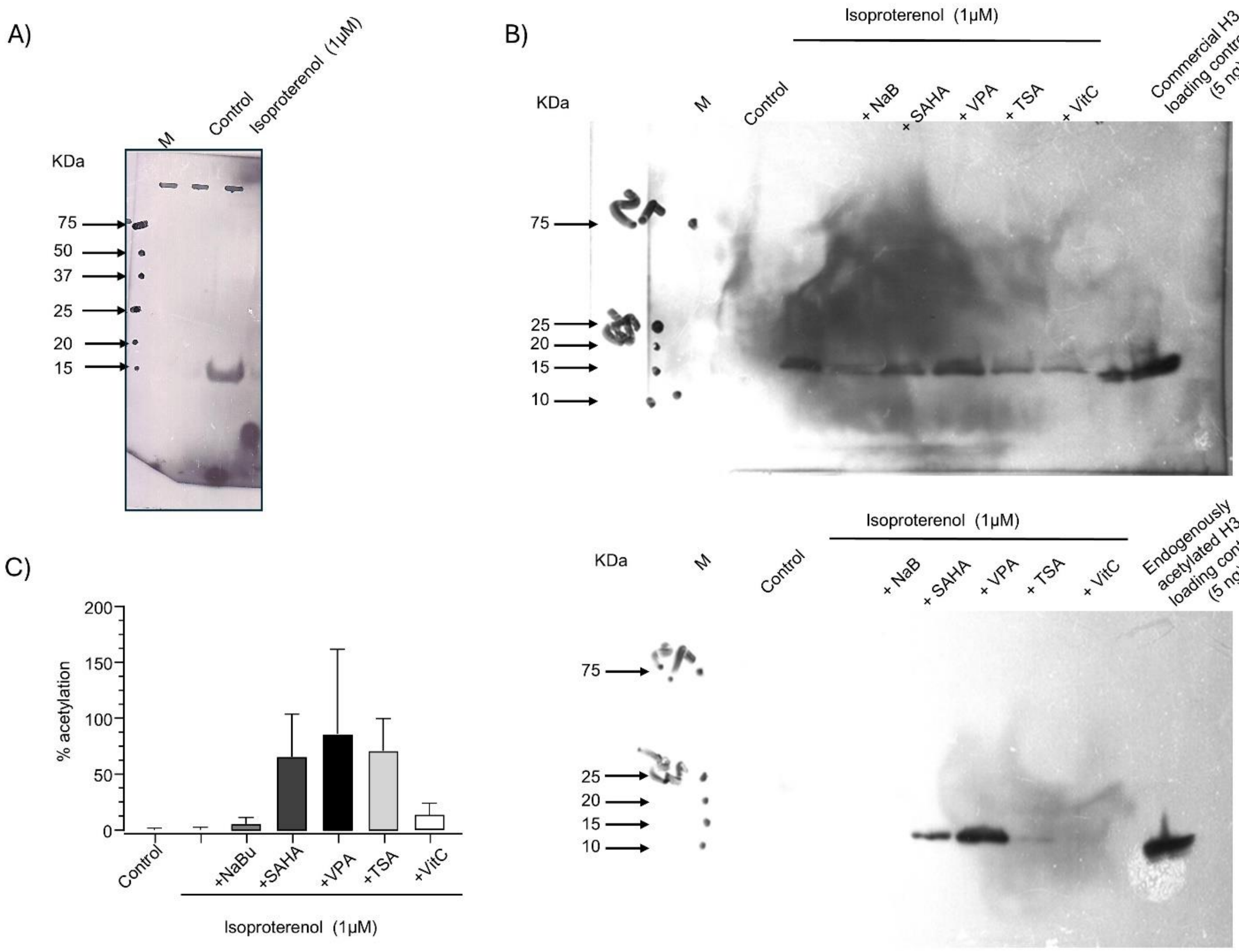

Supplementary Fig. 5. **Media release of endogenous histone H3 from damaged cardiomyocytes and the influence of *in vivo* acetylation on toxicity** Full Western Blots corresponding to Fig 4. A, and C. (A) Western blot image of locally produced endogenous H3 detected in the culture media after 24 h of isoproterenol treatment (1µM) of cultured cardiomyocytes; untreated (Control). (B) Representative western blot images of media H3 (upper) after 24 h without (control) and with isoproterenol treatment (1µM; horizontal bar) and HDAC inhibitors as indicated, and its acetylated form (lower) as detected by anti-H3 acetyl K9+K14+K18+K23+K27 antibody (Abcam, Cambridge, uk). Precision Plus Protein Dual Color Standards (BioRad, Hertfordshire, UK) were used as a marker, labelled as 'M' in both A, B and C. The expected molecular weight for H3 protein is 15 kDa. (C) The calculated % acetylation (n=3; band intensity of acetylated H3/ band intensity of total H3x100).

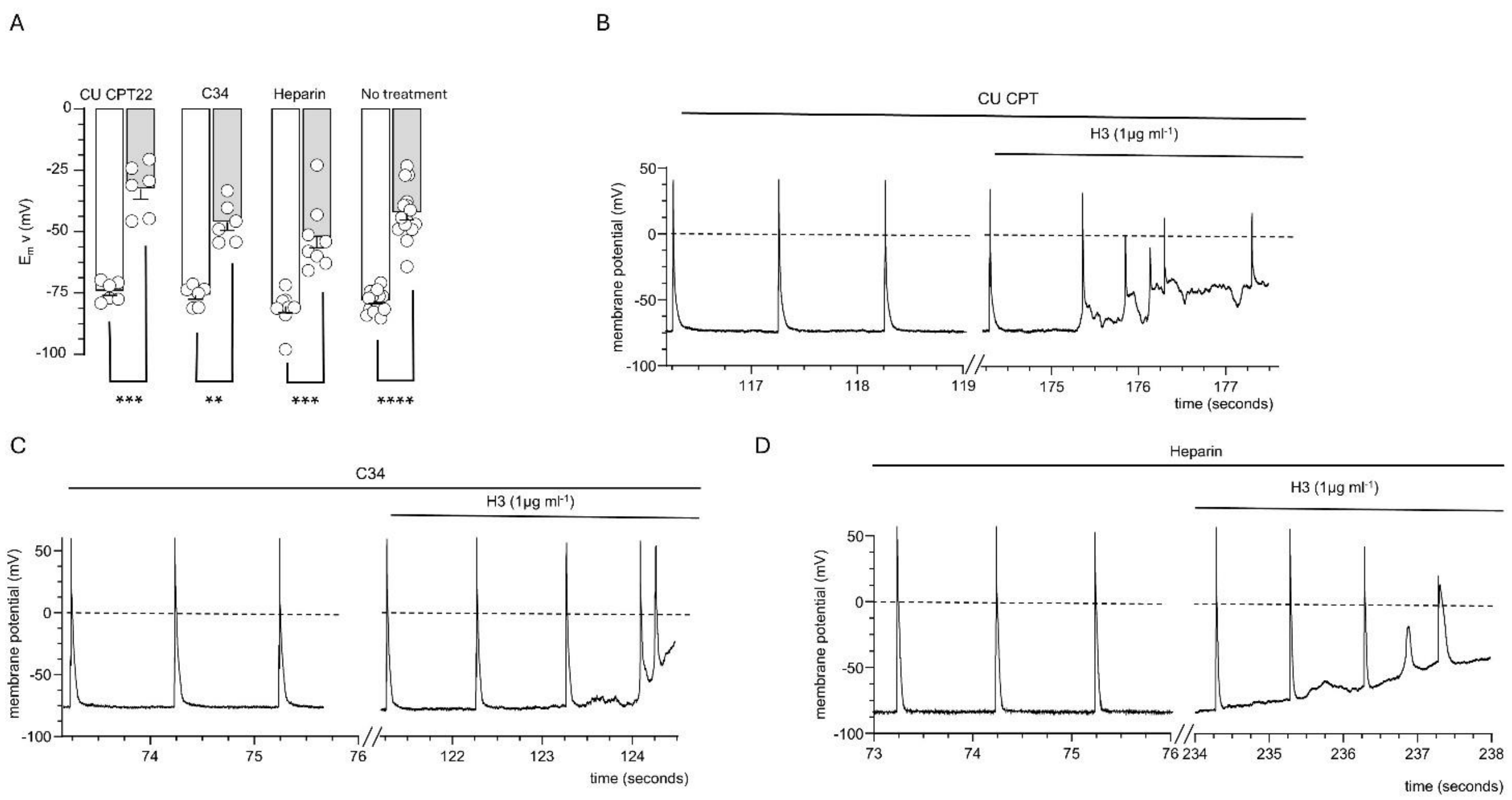

Supplementary Fig. 6. Inhibition of Toll-like receptor TLR2/ TLR4 using CUCP-2250 or C34 inhibitors, or pharmacological neutralization using heparin did not eliminate extracellular histone H3 toxicity. Rat ventricular myocytes were preincubated with each drug at 37°C for 2 hrs, then subjected to whole cell current clamp recording. (A) Summary data (Mean ±SEM) of measured E_m before (control; white) and following H3 ($1\mu g\ ml^{-1}$) superfusion (grey), test conditions is shown above the bar graph, , each circular symbol represents individual data point; P values obtained from a two-tailed paired Student's t-test, ** $P < 0.01$, *** $P < 0.001$, **** $P < 0.0001$. (B-D) Representative trace examples in test conditions described above, drugs were added to the extracellular solution for the duration of experiments to rule out their possible effects (Dashed horizontal line), superfusion of H3 ($1\mu g\ ml^{-1}$) is indicated using solid horizontal line. Slow gradual depolarisation of the membrane potential leading to spontaneous APs, marked with asterisk, and EADs-like oscillations occurred in the presence of each of CUCP-2250, C34 inhibitors, and heparin and were similar to the effects of H3 ($1\mu g\ ml^{-1}$) when examined in untreated cardiomyocytes. H3 induced EAD-like oscillations developed in all cells apart from 2 of the 7 exposed to heparin.

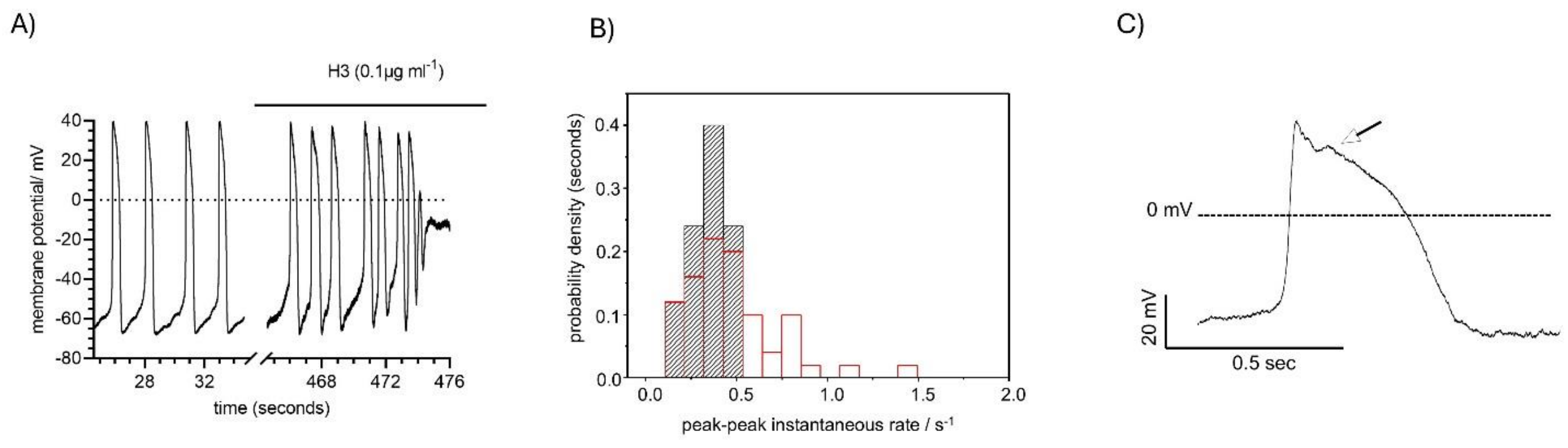

Supplementary Fig. 7. Histone H3 (0.1µg ml^{-1}) destabilise and disrupts human iPSC-derived cardiomyocytes membrane potential. (A) Representative recordings from spontaneously beating hiPSC cardiomyocyte before and during superfusion of H3 (horizontal line). The afterhyperpolarization depolarized from -62.6 ±5.3 during spontaneous activity to a maintained steady level of -13.6 ±7.3 mV (mean ±SEM; n=4). This occurred by 263.2 ±76.9 seconds (mean ±SEM; n=4) following H3 superfusion. This period is not statistically different from that found for rat cardiomyocytes (Fig 1 H; 218 ± 64.0 seconds; p=0.67 unpaired t-test). (B) Histogram of peak to peak instantaneous rate before (black hatched) and during H3 superfusion (red) - the higher rate could be produced by a depolarizing leakage current (C) action potential with small EAD-like event indicated by arrow during H3 superfusion.

A)

Histones destabilise and disrupt rat ventricular myocytes membrane potential in concentration and time dependent manner					
Hearts#	#cells	H3 (μg ml^{-1})			
		0.001	0.01	0.1	1
1	4			2	2
2	1			1	
3	2		2		
4	2		2		
5	1				1
5	5			2	3
6	4		2	1	1
7	6	3		1	2
8	4	1		1	2
9	3	3			
10	4	3			1
11	3		2		1
12	2				2
Total # cells/ experiment	41	10	8	8	15

Hearts#	#cells	H4 (μg ml-1)				
		0.001	0.01	0.1	1	3
13	2			2		
14	2		2			
15	1				1	
16	1					1
17	4				4	
18	2				2	
19	1				1	
20	6	4	1	1		
21	3					3
22	3				1	2
23	2					2
24	3	2			1	
25	2			1	1	
26	3		2	1		
27	3			3		
28	3		3			
29	1					1
Total # cells/ experiment	42	6	8	8	11	9

B)

Histones proteins disrupt intracellular Ca2+ handling in rat ventricular myocytes.										
Hearts#	#cells	H3 (μg ml^{-1})				Hearts#	#cells	H4 (μg ml^{-1})		
		0.01	0.1	1				0.01	0.1	1
30	6		4	2		34	10		4	6
31	6	3	3	1		35	12	1	7	4
32	11	1	5	5		36	16	7	5	4
33	10	3	6			37	15	7	6	2
Total # cells/experiment	33	7	18	8		Total # cells/experiment	53	15	22	16

C)

H3 induction of non-specific leak and membrane damage			
Hearts#	# cells	inhibitors + H3 (0.1 μg ml^{-1})	inhibitors
38	3	2	1
39	4	2	2
40	6	3	3
Total # cells/experiment	13	7	6

D)

Media release of endogenous histone H3 from damaged cardiomyocytes and the influence of in vivo acetylation on toxicity.							
Hearts#	# cells	ISO only	ISO+NaBu	ISO+SAHA	ISO+VPA	ISO+TSA	ISO+VitC
41	3	1		2			
42	3	1		2			
43	3	1		2			
44	3	1		2			
45	3		3				
46	2	1	1				
47	5	2					3
48	4	2					2
49	3	1					2
50	3	1					2
51	5	1			4		
52	5	1			4		
53	4	1			3		
54	5	1	1			3	
55	4	1				3	
56	4	1	1			2	
Total # cells/experiment	59	17	6	8	11	8	9

E)

Targeted In Vitro Acetylation of Histone H3 reduces toxicity				
Hearts#	# cells	Recombinant PanAcetylated H3	H3+LG2_1	H3+Pan_acetyle Ab
57	2	2		
58	3	3		
59	4	4		
60	2		1	1
61	2		1	1
62	4		2	2
63	2		1	1
64	2		1	1
65	2		1	1
Total # cells/experiment	23	9	7	7

Supplementary Tables. 1. Summary table showing biological replicates used, the total number of cells per experiment. (A) Histones destabilise and disrupt rat ventricular myocytes membrane potential in concentration and time dependent manner. (B) Histones proteins disrupt intracellular Ca^{2+} handling in rat ventricular myocytes. (C) H3 induction of non-specific leak and membrane damage. (D) Media release of endogenous histone H3 from damaged cardiomyocytes and the influence of *in vivo* acetylation on toxicity. (E) Targeted *In Vitro* Acetylation of Histone H3 reduces toxicity.
